# A Systematic Review of Aminaphtone from Pathophysiology to Clinical Applications: Focus on New Rheumatological Acquisitions

**DOI:** 10.3390/ph16040569

**Published:** 2023-04-10

**Authors:** Emanuele Gotelli, Stefano Soldano, Elvis Hysa, Greta Pacini, Carmen Pizzorni, Sabrina Paolino, Maurizio Cutolo, Alberto Sulli

**Affiliations:** Laboratory of Experimental Rheumatology, Academic Division of Clinical Rheumatology, Department of Internal Medicine, University of Genova, IRCCS San Martino Polyclinic Hospital, 16132 Genova, Italy

**Keywords:** Aminaphtone, blood perfusion, Raynaud’s phenomenon, systemic sclerosis, vascular molecules

## Abstract

Aminaphtone is a chemical drug that has been used for more than thirty years to treat a variety of vascular disorders, with good clinical results and a satisfying safety profile. In the last two decades, multiple clinical studies have reported the efficacy of the drug in different clinical scenarios of altered microvascular reactivity, describing the downregulation of adhesion molecules (i.e., VCAM, ICAM, Selectins), vasoconstrictor peptides (i.e., Endothelin-1), and pro-inflammatory cytokine expression (i.e., IL-6, IL-10, VEGF, TGF-beta) by Aminaphtone. In this review, we summarize the current knowledge concerning Aminaphtone, with particular attention to rheumatological conditions in which microvascular disfunction plays a pivotal role, such as Raynaud’s phenomenon and systemic sclerosis. These latter conditions may represent a promising field of application for Aminaphtone, due to the growing pre-clinical, clinical, and instrumental reports of efficacy. However, randomized, double-blind, placebo-controlled clinical trials are lacking and are desirable.

## 1. Introduction

Aminaphtone (3-Methyl-1,4-dioxo-1,4-dihydro-naphthalen-2-y 4-amino-benzoate) is a chemical compound (molecular formula: C_18_H_15_NO_4_) that is licensed as an endothelial protector for “capillary disorders” and classically chronic venous insufficiency [1,2]. Over the past two decades, several pre-clinical studies have reported that Aminaphtone can interfere with a broad spectrum of biological mediators involved in the regulation of endothelial homeostasis, such as vasoconstrictor and vasodilator molecules as well as pro-inflammatory and pro-fibrotic cytokines [3]. Thus, there is an emerging body of spontaneous clinical reports on the use of Aminaphtone in various clinical conditions, such as idiopathic cyclic edema syndrome [4], minor bleeding disorders [5], diabetic nephropathy [6], and Raynaud’s phenomenon (RP) (both primary and secondary to systemic sclerosis, SSc) [7].

This review collects the pre-clinical and clinical reports on Aminaphtone, grouped by target of action and disease, focusing on rheumatological conditions, whose pathophysiological background has been resumed with regard to the potential role of Aminaphtone in their management.

## 2. Search Strategy and Literature Results

A systematic literature search concerning Aminaphtone was electronically performed in PubMed and Crossref databases, according to the Preferred Reported Items for Systematic Reviews and Meta-Analyses (PRISMA) statement checklist, up to 31 December 2022 [8].

The review was registered in Open Science Framework (registration DOI: https://doi.org/10.17605/OSF.IO/DM3C9 (accessed on 24 March 2023).

The terms “aminaftone” and “aminaphtone” were chosen for the search, and all manuscripts (English and Italian language) containing them were considered for the review.

Two authors (E.G. and A.S.) independently identified, reviewed, and analyzed the manuscripts (original articles, case series, case reports, narrative or systematic reviews). Book chapters and conference abstracts were excluded from the review.

Twenty-eight articles whose full text was available were identified (English and Italian language). Among these articles, only one randomized controlled trial (RCT) was available; the other papers were pre-clinical and open-label clinical studies, including case reports. The main clinical studies are listed in Table 1, which also specifies the diseases in which this molecule is used, together with the related subjective and objective evidence of drug efficacy.

## 3. Results of the Systematic Review

**Figure 1:**
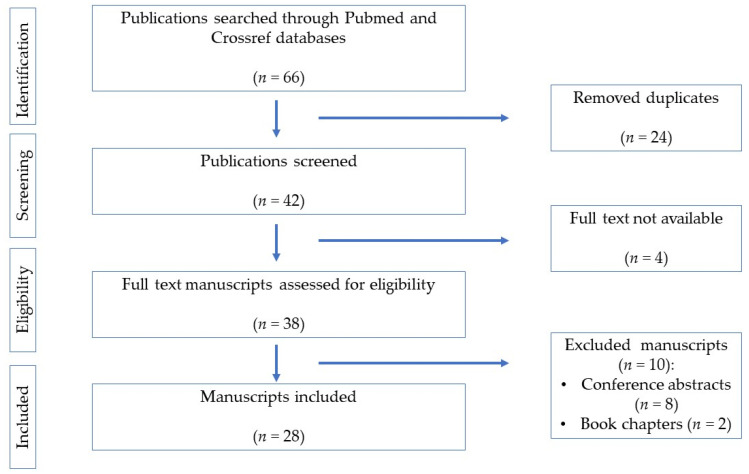
Flowchart of the systematic review according to PRISMA guidelines.

Of the 38 screened manuscripts, 28 were included in our systematic review. The flowchart of the process is depicted in Figure 1.

The 28 selected papers were further categorized according to the different steps of pathophysiology of microvascular damage that Aminaphtone seems to antagonize: recruitment of leukocytes and platelets through overexpression of adhesion molecules, overexpression of endothelin-1 (ET-1), and overexpression of specific cytokines and chemokines.

Consequently, the diseases in which Aminaphtone has been used are described together with related pathophysiology in the following paragraphs.

## 4. Downregulation of Endothelial Cell Adhesion Molecules and Chronic Venous Insufficiency

The vascular endothelium is a dynamic tissue between the vessel wall and blood stream that reacts to a large plethora of stimuli (humoral, neural, hemodynamic). Endothelium can synthetize and release vasodilator (e.g., nitric oxide, prostacyclin) and vasoconstrictor (e.g., ET-1, angiotensin-II) molecules according to different situations [9]. When the endothelium is damaged, there is an over-expression of endothelial cell adhesion molecules and selectins, including vascular cell adhesion molecule-1 (VCAM-1), intercellular adhesion molecule-1 (ICAM-1), E-selectin (also known as ELAM-1), L-selectin, and P-selectin. These molecules promote platelet aggregation and adhesion of leukocytes from the blood stream, which migrate across the endothelium, triggering local and/or systemic inflammation [10].

Chronic venous insufficiency is a common disease with multifactorial etiology. The first step of the pathological process is a vascular endothelium dysfunction with an over-expression of adhesion molecules. The venous stasis causes hypoxia and ischemia, with activation of fibroblasts and hyper-production of vasoconstrictor molecules, up to the onset of venous ulcers [11].

De Anna et al. reported the phlebotonic, lymphagogue, anti-sludge, and oncotic properties of Aminaphtone in a cohort of patients with chronic venous insufficiency. In fact, the drug enhanced wall resistance of veins, capillaries, and lymphatic vessels [2]. These clinical observations may be explained at a molecular level by the study of Scorza et al., who reported in a group of 12 SSc patients treated with Aminaphtone a significant reduction of ELAM-1 and VCAM-1 serum concentrations (see the dedicated section of this review for further details) [12].

Moreover, Belczak et al. compared the effectiveness of different veno-active drugs for chronic venous disease (Aminaphtone, bioflavonoids, coumarin) in a randomized, double-blind, placebo-controlled trial (RCT) [13]. They assigned Aminaphtone to 36 patients and evaluated the reduction of limb volume and improvements of tibio-tarsal range of motion and quality of life. Aminaphtone significantly improved quality of life, also providing relief from edema, pain/burning, pruritus/paresthesia, and heaviness/fatigue of affected limbs. Only one patient stopped the drug due to an adverse event (headache) [13].

In 2015, Bentivegna et al. reported the case of a patient with chronic osteomyelitis of the left tibial plateau and ulceration of the overlying skin, which was successfully treated with a combination therapy of antibiotics, prostaglandins, anticoagulants, and Aminaphtone, suggesting a complementary role of the drug in ulceration healing [14].

Finally, the Cochrane Vascular Group recently confirmed a potential role for Aminaphtone as a phlebotonic agent in the 2016 and 2020 update of their systematic review on the topic (“Phlebotonics for venous insufficiency”) [15,16].

## 5. Downregulation of Endothelin-1 and Diabetes

ET is a 21-amino-acid-long peptide produced by the vascular endothelium, vascular smooth muscle cells, and macrophages. These cells release pre-pro-endothelin-1 (PPET-1), which is cleaved to pro-endothelin, an inactive form, and then cleaved to ET by endothelin-converting enzymes. There are three isoforms of ET: ET-1, ET-2, and ET-3. ET interacts with two G-protein coupled receptors, endothelin receptor A (ET-A) and endothelin receptor B (ET-B). ET-A is mainly expressed on vascular smooth muscle, and its activation causes vasoconstriction; on the contrary, ET-B is mainly expressed on the endothelium with vasodilators effects. ET-A has the strongest affinity to ET-1, with vasoconstrictor, pro-fibrotic pro-oxidative, and pro-inflammatory effects [17]. Of note, ET-1 promotes collagen production by human mesangial cells with a pro-fibrotic action at the renal level [18]: this mechanism is involved in the development of microangiopathic disorders such as diabetic nephropathy. Aminaphtone seems to interfere with this pathogenetic pathway, downregulating in vitro PPET-1 gene expression, in a dose- and time-dependent manner, decreasing the final production of ET-1 [19].

Romano et al. supported this observation with the case-report of a patient suffering from type I diabetes and early renal involvement. Aminaphtone significantly ameliorated microalbuminuria in addition to standard therapy after two months of treatment; the positive effect for the patient was lost two months after drug suspension [6].

Finally, an excessive release of ET-1 by endothelial cells after foam sclerotherapy has been reported to be involved in the pathogenesis of benign visual and neurological disturbances [20]. Aminaphtone significantly reduced ET-1 release in murine models after sclerotherapy, but no data are available in humans [21].

## 6. Downregulation of Vascular Endothelial Growth Factor and Edematous Syndromes

When the vascular endothelium is injured, hypoxia stimulates the production of vascular endothelial growth factor (VEGF) by endothelial cells. VEGF promotes angiogenesis, but its over-expression increases vascular permeability, weakening vascular endothelial cadherins adhesion [22]. Felice et al. investigated the effect of Aminaphtone on vascular permeability in vitro and demonstrated a significant protective role of the drug on VEGF-induced degradation of endothelial cadherins [23]. This observation was confirmed by Salazar et al., who analyzed the effects of Aminaphtone on gene expression and production of different chemokines and cytokines by human ECV304 cells (an in vitro surrogate of endothelial cells) after incubation with interleukin (IL)-1β. Aminaphtone down-regulated the expression of a wide range of cytokines at the gene level, such as monocyte chemotactic protein-1 (MCP-1), granulocyte-macrophage colony-stimulating factor (GM-CSF), granulocyte colony-stimulating factor (G-CSF), interferon (IFN)-α, tumor necrosis factor (TNF)-α, IL-1 receptor antagonist, IL-6, IL-7, IL-8, IL-10, IL-15, epidermal growth factor (EGF), chemochin ligand (CXCL)-6, transforming growth factor (TGF)-β2, and VEGF [3,24].

Moreover, the protective role of Aminaphtone on endothelial cadherins may explain the positive effects of the drug on microcirculation disorders, such as idiopathic cyclic edema syndrome. This syndrome is characterized by capillary hyperpermeability, with interstitial retention of fluid that causes edema with daily variations of body weight [25]. De Godoy et al. demonstrated that Aminaphtone could reduce not only the intensity of the edema but also the accompanying symptoms, such as hypnic headache, stasis dermatitis, cellulitis, and lower limb lymphedema [4,26,27,28,29,30].

Aminaphtone has been also used in traumatic injuries to relieve associated symptoms (edema, swelling, and pain) [31] and in cystoid macular edema following cataract surgery to improve visual acuity and decrease central foveal thickness [32].

Moreover, Aminaphtone has been employed as adjuvant therapy in minor bleeding [5,33] and in purpuric disorders, such as Schamberg’s disease [34].

## 7. Microvascular Dysfunction, Systemic Sclerosis, Secondary Raynaud’s Phenomenon, and Pulmonary Arterial Hypertension (PAH)

The above-reported observations have promoted great interest concerning the use of Aminaphtone in those autoimmune rheumatic diseases characterized by endothelial impairment and microvascular dysfunction, including SSc.

Microangiopathy is the driver of SSc pathophysiology, in addition to alterations of innate and adaptive immune response and fibrosis of skin and internal organs [35,36]. Microangiopathy can be easily detected by nailfold videocapillaroscopy (NVC), an imaging technique that is able to assess the morphological structure of peripheral capillaries at the level of the periungual bed of the hands [37]. SSc microvascular damage can be classified into three different capillaroscopic patterns, “Early”, “Active”, and “Late”, which reflect the evolution of the disease and correlate with the progressive severity of SSc organ involvement [38,39].

In SSc, several triggers cause endothelial damage, promoting early pathological activation of the vascular endothelium with over-expression of adhesion molecules [40]. Interestingly, in a randomized open-label study, Scorza et al. administered Aminaphtone to 12 consecutive SSc patients in addition to standard background vasodilatory therapy (calcium channel blockers and cyclic intravenous iloprost). They reported a significant reduction of ELAM-1 and VCAM-1 serum concentrations after 12 weeks of treatment compared with 12 consecutive SSc patients in the control group who had not received Aminaphtone in addition to standard vasodilatory therapy [12].

A damaged vascular endothelium also produces a large amount of ET-1, which induces fibroblast proliferation and the switch from naïve (M0) to anti-inflammatory alternatively activated (M2) macrophages, with a cascade of pro-fibrotic molecules that stimulate collagen synthesis [41,42]. Furthermore, the strong vasoconstrictor effect of the ET-1 peptide contributes to one of the most evident manifestations of SSc microangiopathy, the Raynaud’s phenomenon (RP) [43]. RP is the result of an unbalanced ratio between vasoconstrictor and vasodilator molecules at the level of small-caliber muscle arteries, arterioles, and capillaries, with vasospasm of digital blood vessels (toes, nose, nipples, and tongue can be also affected). RP consists of two or three phases, characterized by a sequence of vasospasm/ischemia (blanching phase), tissue hypoxia/anoxia (cyanotic phase), and reperfusion (hyperemic phase). RP has been classified into two forms: a primary idiopathic form, in which vasospasm is only an exasperated response to physiological stimuli (cold, anxiety, intense emotions), and a secondary form, in which vasospasm is the result of pathological microvascular damage, such as in SSc [44].

The drugs currently licensed for the treatment of SSc-related active RP are calcium-channel blockers, phosphodiesterase type 5 inhibitors, and intravenous iloprost. However, the treatment of secondary RP is often unsatisfying, and the use of these drugs is limited by frequent side effects (hypotension, peripheral edema, headache), high costs, and hospital setting for administration [45,46,47].

Hence, Aminaphtone has also been tested for the management of SSc-related RP. In 2015, Parisi et al. enrolled 108 SSc patients, dividing them in two groups: one group was treated with the standard of care for the control of RP (calcium channel blockers, intravenous iloprost, ET receptor antagonist, and phosphodiesterase type 5 inhibitors), and the second group with the standard of care together with Aminaphtone [48]. After 48 weeks of treatment, the group of patients also treated with Aminaphtone reported a significant reduction of number of RP attacks (*p* = 0.02), as evaluated by Raynaud’s Condition Score (RCS) and by Visual analogue scale (VAS) of pain (*p* = 0.04 and *p* = 0.04, respectively) as compared with the standard of care group, without relevant side effects [48].

Ruaro et al. reported the case of a patient with RP secondary to SSc and a history of scleroderma digital ulcers; the administration of Aminaphtone in combination with standard treatment (ET receptor antagonist–bosentan) for 4 weeks improved the symptoms related to RP, as evaluated by RCS and frequency and duration of RP attacks, as well as peripheral blood perfusion, evaluated at the level of the hands by Laser speckle contrast analysis (LASCA) [49].

In 2019, a new study confirmed previous observations in a larger cohort of patients [7]. Thirty-five SSc patients were recruited and received Aminaphtone for active secondary RP management in addition to ongoing treatments, with the exclusion of intravenous prostanoids, ET receptor antagonists, and phosphodiesterase type 5 inhibitors. The amelioration of RP clinical symptoms, assessed by RCS and other RP parameters (frequency and duration), was also confirmed by the improvement of skin blood perfusion, assessed by LASCA, at up to six months of treatment. A progressive statistically significant increase of peripheral blood perfusion as well as a progressive statistically significant decrease of RCS as well as frequency and duration of daily RP attacks were observed from baseline to week 12 in all skin areas of the hands. Neither further increase of skin blood perfusion nor reduction of RCS was observed from week 12 to week 24; five weeks after Aminaphtone discontinuation, peripheral blood perfusion values were still significantly higher than those at baseline in most skin areas, and clinical efficacy was sustained. Serious adverse events were not observed during the study. Of note, the results concerning clinical efficacy were similar in patients with “Early”, “Active”, and “Late” capillaroscopic patterns of nailfold microangiopathy, as well as in patients with limited (including CREST syndrome-calcinosis, Raynaud phenomenon, esophageal dysfunction, sclerodactyly, and telangiectasia) and diffuse cutaneous SSc, underlining the influence of the drug in all microvascular phases and clinical subsets of the disease [7].

Of relevance, several authors have reported satisfactory tolerability of Aminaphtone, although manuscripts regarding the long-term safety of the drug are lacking. Although we decided to eliminate conference abstracts from our systematic review, we report only a preliminary work in this regard [50]. Seventy SSc patients with symptomatic secondary RP were treated with Aminaphtone (average 75 milligrams twice a day) in addition to concomitant vasodilating therapy, including ET receptor antagonists and intravenous iloprost [50]. Improvement of RCS was clinically significant with Aminaphtone treatment, and the drug showed a good tolerability and safety profile (no changes in routine blood tests). The only adverse event reported was headache (8.6% of cases), and no patient discontinued treatment due to intolerance during a four-year follow-up [50].

Finally, an excess of circulating ET-1 is also involved in the pathogenesis of SSc-related pulmonary arterial hypertension (PAH). PAH occurs when the vascular remodeling causes an irreversible fibrosis of lung arterioles with increased resistance in pulmonary circulation, which leads to right ventricular failure [51]. Aminaphtone has been studied in a murine model of monocrotaline-induced pulmonary hypertension. The drug decreased the mortality of rats after 5 weeks of treatment, likely reducing ET-1-induced right ventricular hypertrophy and remodeling [52]. ET receptor antagonists, such ambrisentan, bosentan, and macitentan, are currently the only drugs targeting the ET pathway to be approved for the treatment of SSc-related PAH [53,54], and clinical studies regarding the possible use of Aminaphtone in SSc-PAH in humans are currently lacking.

## 8. Primary Raynaud’s Phenomenon

As above discussed, primary RP (PRP) is a benign condition, characterized by an excessive acral vasospasm that reduces cutaneous blood flow, without association to pathological conditions [44]. Although benign, this condition can be disabling due to the frequency and intensity of attacks. Calcium channel blockers have demonstrated minimal efficacy in the symptomatic treatment of PRP, while alternative drugs are not currently available [55]. Different vasodilators (angiotensin-converting enzyme inhibitors, alpha-blockers, prostaglandin/prostacyclin analogues, thromboxane synthase inhibitors, selective serotonin reuptake inhibitors, nitrate or nitrate derivatives, phosphodiesterase inhibitors) have been systematically compared to placebos, but no strong evidence of efficacy has been reported [56].

For this reason, in 2019, Ruaro et al. tested Aminaphtone for the treatment of PRP in an unblinded setting [7]. Eleven patients with PRP were recruited and treated with Aminaphtone 75 mg twice per day for six months (the concomitant use of calcium channel blockers was not permitted). The drug increased peripheral blood perfusion, as evaluated by LASCA, improved RCS, and reduced frequency and intensity of vasospastic attacks, in comparison with a control group [7].

As observed in patients with secondary RP, five weeks after treatment discontinuation, peripheral blood perfusion values were still significantly higher than those at baseline in most skin areas, and clinical efficacy was sustained [7].

Furthermore, some patients with “puffy fingers” (pathological swelling of the fingers that can occur in the course of SSc) reported an improvement of symptoms, up to complete resolution, during treatment with Aminaphtone [7]. This was possibly related to the reduction of the edematous clinical phase of “puffy fingers” [2,12,32].

## 9. Discussion

Aminaphtone is an established drug with current growing interest in the rheumatological field.

Regarding its pharmacokinetic properties, after oral intake, Aminaphtone is partially metabolized to phthiocol and eliminated through the urine within 72 h, with the maximum excretion level about 6 h after administration. Concerning preclinical safety data, the tests of acute toxicity (four animal species for doses up to 3 g/kg), subacute toxicity (two animal species up to 100 mg/kg, for 90 days) and chronic toxicity (50 mg/kg in dogs for 280 days) showed no symptoms of tissue lesions or changes in organ functions. Aminaphtone also had no teratogenic or mutagenic effects [7]. To date, Aminaphtone has proven to be a versatile and useful molecule for the treatment of peripheral vascular disorders; characteristics of the main clinical studies are listed in Table 1.

**Figure 2:**
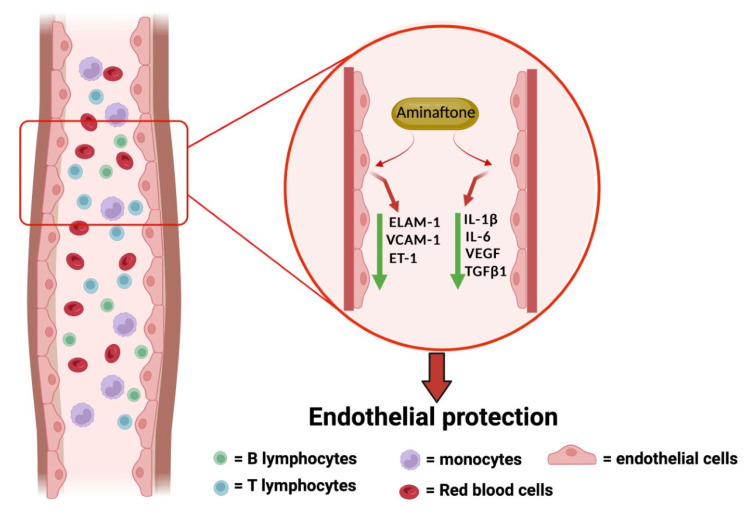
Suggested biological effects of Aminaphtone. Produced at www.biorender.com (accessed on 7 April 2023).

From a rheumatological point of view, the interest in Aminaphtone is growing in the treatment of RP (both primary and secondary to SSc) due to both the promising evidence regarding the mechanism of action of the molecule, as summarized in Figure 2, and the unsatisfactory current therapeutic options.

PRP is a disabling condition, with episodes of acral pallor or cyanosis, associated with symmetrical pain and numbness of the extremities, typically triggered by cold, without evidence of digital pitting, ulceration, gangrene, or association with an underlying disease [44]. Patients with PRP should be carefully investigated by rheumatologists to identify elements of suspicion for the presence or risk of developing of connective tissue diseases [57]. In addition to this, therapy for PRP is often required by patients, and calcium channel blockers have poor tolerability. Moreover, the evaluation of the efficacy of treatments for PRP is challenging; in fact, even the most recent Cochrane systematic review highlighted that the outcomes of clinical studies in this regard are only subjective (frequency, severity, and duration of vasospastic attacks; RCS; and quality of life). Aminaphtone showed promising effects on PRP, both subjective and objective, as reported by Ruaro et al., who described an objective increase of peripheral blood perfusion by LASCA after treatment [7].

A secondary RP is present in virtually all patients with SSc and is associated with the development of pitting, digital ulcers, or gangrene [58]. Patients with SSc often need to escalate vasodilatory treatment due to these complications, although this approach is not always well tolerated [59]. Aminaphtone could be an interesting complementary therapy to concomitant vasoactive drugs, and two open-label studies have reported significant improvements in perfusion, both subjective and objective (LASCA) [7,47].

Of note, this review has some limitations. Only one RCT study is available (Aminaphtone use in the treatment of chronic venous insufficiency). The clinical studies included and analyzed in this review on Aminaphtone and RP (both primary and secondary) are non-randomized and focused on single-center cohorts of patients (only in Italy and Brazil). It was not possible to perform a meta-analysis of data collected due to their heterogeneity. Finally, most clinical efficacy outcomes for Aminaphtone are subjective measures. New tools, such as LASCA and ultrasound quantitative assessment, are being studied to better characterize the vascularity of digital fingers of patients, in particular in the course of SSc, and could be used also to objectively investigate the efficacy of vasodilators/vasoprotector drugs [60].

## 10. Conclusions

Aminaphtone shows various interesting mechanisms of action (interference with overexpression of adhesion molecules, ET-1, pro-inflammatory cytokines, VEGF) that go beyond a generic antagonism of microvascular dysfunction, along with excellent tolerability. As recently observed, Aminaphtone may play a role in the management of both primary and secondary RP, with a good safety profile. RCTs are needed to better investigate Aminaphtone’s efficacy and to confirm the potential “endothelial protector” role of the drug.

## Figures and Tables

**Table 1:** Main clinical studies regarding Aminaphtone (LASCA = Laser Speckle Contrast Analysis; RCS = Raynaud’s condition score; RP = Raynaud’s phenomenon; SD = standard deviation; SSc = Systemic sclerosis; VAS = visual analogue scale).

	Consoli (30)	De Anna (2)	De Godoy (4)	Scorza (12)	De Godoy (28)	Belczak (13)	De Godoy (32)	Parisi (47)	Ruaro (7)
Country	Italy	Italy	Brazil	Italy	Brazil	Brazil	Brazil	Italy	Italy
Year	1985	1989	2008	2008	2011	2013	2014	2015	2019
Enrolled patients, *n*	42	66	15	24	82	136	30	108	92
Age range or mean age (years ± SD)	20–70	53 ± 11	22–49	53 ± 14	18–58	51	13	52	59
Disease investigated	Traumatic injuries	Chronic venous insufficiency	Idiopathic cyclic edema syndrome	SSc-related RP	Cellulite and idiopathic edema syndrome	Chronic venous insufficiency	Gingival bleeding	SSc-related RP	Primary and SSc-related RP
Aminaphtone patients, *n*	42	66	15	12	82	36	15	57	46
Control patients, *n*	No	48	No	12	No	57	No	51	46
Placebo patients, *n*	No	No	No	No	No	43	15	No	No
Methods of evaluation	Semiquantitative	Semiquantitative and quantitative	Quantitative	Quantitative	Quantitative	Semi-quantitative and quantitative	Qualitative (yes/no)	Semi-quantitative and quantitative	Semi-quantitative and quantitative
Subjective measures	Tumefaction, range of movements, spontaneous and or provoked pain	Itching, venous pain, night cramps, paresthesia	/	/	/	Quality of life	/	Pain VAS, RCS, tingling, numbness	RCS, Raynaud frequency and duration
Objective measures	/	Circumference of ankle, district venous pressure	Lower limb volumetry (mL)	Serum concentration of ECAM-1, VCAM-1, and ICAM-1	Weight	Mean limb volumetry, tibio-tarsal joint range of movements	Intraoral clinical examination	Average attacks RP (n°/day)	Skin blood perfusion (LASCA)
Posology (mg per day)	150–225	300–450	225	225	225	150	150	150	150
Duration of treatment	6–30 days	90 days	5 days	12 weeks	3 days	30 days	5 days	12 months	24 weeks
Concomitant therapy	Yes	Yes	Yes	Yes	Not specified	No	No	Yes	Yes
Efficacy	Yes	Yes	Yes	Yes	Yes	Yes	Yes	Yes	Yes
Side effects	Heartburn (6 pts), skin rash (1 pt)	Heartburn (2 pts)	Not reported	Not reported	Not reported	Headache (1 pt)	Not reported	None	Headache (2 pts)

## Data Availability

Data sharing is not applicable.
